# Ochratoxin A induces endoplasmic reticulum stress and fibrosis in the kidney via the HIF-1α/miR-155-5p link

**DOI:** 10.1016/j.toxrep.2023.01.006

**Published:** 2023-01-18

**Authors:** Seon Ah Yang, Kyu Hyun Rhee, Hee Joon Yoo, Min Cheol Pyo, Kwang-Won Lee

**Affiliations:** a Department of Biotechnology, College of Life Sciences and Biotechnology, Korea University, 02841 Seoul, the Republic of Korea; b Department of Food Bioscience and Technology, College of Life Sciences and Biotechnology, Korea University, 02841 Seoul, the Republic of Korea

**Keywords:** Ochratoxin A, HIF-1α, MiRNA-155–5p, ER stress, Fibrosis, Kidney

## Abstract

Ochratoxin A (OTA) is a ubiquitous fungal toxin found in agricultural products and foods that is toxic to both humans and animals. OTA mainly affects kidney, but the mechanisms underlying OTA-induced nephrotoxicity remain not fully understood. MicroRNA (miRNA) is involved in key cellular processes. The toxic mechanism and regulatory effects of miRNAs on OTA toxicity in kidney, and particularly the role of HIFα-1/miR-155–5p on OTA-caused ER stress and fibrosis, were investigated in this study. OTA induced hypoxia-like conditions such as ER stress and fibrosis in HK-2 cells and renal tissues via modulating HIF-1α, which was followed by regulation of ER stress-related proteins (GRP78 and ATF-4), as well as fibrosis-related markers (fibronectin, α-SMA, and E-cadherin). Notably, a total of 62 miRNAs showed significant differential expression in kidney of OTA-treated mice. Under OTA exposure, HIF-1α enhanced miR-155–5p expression, causing ER stress and fibrosis in HK-2 cells. HIF-1α knockdown decreased OTA-induced miR-155–5p expression as well as ER stress and fibrotic responses, whereas miR-155–5p overexpression restored this. Our data suggest that OTA enhances ER stress and fibrosis in the kidney through upregulating the HIF-1α/miR-155–5p link.

## Highlights

• Ochratoxin A increases hypoxia, endoplasmic reticulum stress, and fibrosis in kidney.
• OTA treatment regulates a total of 62 miRNAs in mouse renal tissues.
• OTA induces miR-155–5p upregulation contributing ER stress and fibrosis in kidney.
• HIF-1α is responsible for the miR-155–5p induction under OTA exposure in kidney.

## Graphical Abstract

**Figure:**
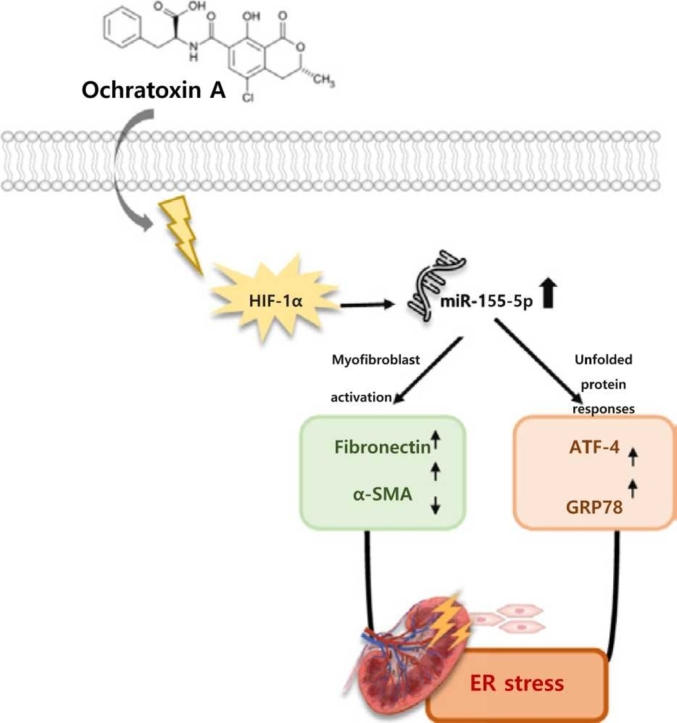

## 1 Introduction

Ochratoxin A (OTA), a secondary toxic metabolites generated by the *Aspergillus* and *penicillium* species, is one of the most common mycotoxins found in foods such as nuts, cereals, coffee beans, and fermented tea [1]. Because of widespread exposure, humans are at risk of OTA-induced adverse effects after consuming contaminated foods containing OTA [2]. According to the International Agency for Research on Cancer, OTA is categorized as group 2B carcinogen, and the European Food Safety Agency has set up benchmark dose lower confidence (BMDL_10_) of 14.5 μg OTA/kg body weight (bw) per day accounting neoplastic effects [3], [4]. Furthermore, due to its binding to plasma albumin and low rate of metabolism in the body, OTA has a relatively long half-life, potentially leading to accumulation [4]. It exhibits a variety of toxicities, including genotoxicity, immunotoxicity, neurotoxicity, and nephrotoxicity [5]. Multiple locations along the nephron are affected by OTA, with the kidney being the principal target [6].

Chronic kidney disease (CKD) is a persistent renal damage defined as a progressive and irreversible loss in kidney functions over time [7]. Chronic hypoxia and endoplasmic reticulum (ER) stress were discovered to be shared mechanisms that contribute to the advancement of CKD [8]. Hypoxia is a pathologic condition associated with insufficient oxygen supply to support metabolism [9]. The kidney is assumed to be sensitive to hypoxia due to the arteriovenous oxygen shunt. The ER is the main organelle involved in proteostasis, including protein folding, protein synthesis, and the degradation of misfolded proteins [10]. During hypoxia, protein metabolism is disrupted, resulting in the buildup of unfolded proteins via ER dysfunction, which is known as ER stress [8]. The progression of CKD is an irreversible process that involves broad fibrotic changes that eventually lead to end-stage renal failure [11]. As a response of several forms of tissue damage in the kidney, extensive interstitial myofibroblast activation and abnormal buildup of extracellular matrix (ECM) developed throughout the progression of renal fibrosis. The deposition of fibrotic matrix inhibits kidney function, eventually leading to renal failure [12], [13].

As a key transcription factor, hypoxia inducible factor-1 (HIF-1α) regulates hypoxia [14], [15]. Meanwhile, hypoxia is known to induce ER stress and to be an important determinant of renal fibrosis [8], [16]. The interaction between hypoxia, oxidative stress, and ER stress has been established: Hypoxia-induced reactive oxygen species (ROS) disrupt redox homeostasis in the ER, promoting ER stress [10], [17]. Several studies have shown that elevated HIF-1α expression promotes fibrogenesis by accelerating epithelial-to-mesenchymal transition (EMT) in a variety of organs [14], [18]. However, the underlying mechanism by which HIF-1α promotes these pathophysiological damages in kidney under OTA exposure remains to be elucidated.

MicroRNA (miRNA) is a type of noncoding, small RNA containing 19–22 nucleotides, which regulates protein-coding gene expression via mRNA degradation or translational repression [19]. It has been discovered that miRNA directly modulates the unfolded protein response (UPR) signaling associated with ER stress and may be a modulator of fibrotic responses in CKD patients with pathological conditions [20], [21]. Albumin-induced miR-4756, for example, has been shown to enhance renal ER stress and EMT in diabetic kidney disease [22]. It was demonstrated that miR-185–5p inhibited kidney fibrosis through suppressing ER stress-responsive protein ATF6 [23]. Meanwhile, miR-155–5p, a hypoxia-inducible miRNA has been linked to ER-stress-induced cardiomyocyte apoptosis and the promotion of renal interstitial fibrosis in obstructive nephropathy [24], [25]. Notably, Bruning et al. [26] revealed that HIF-1α could be responsible for miR-155–5p induction under hypoxia in colon cancer cells, forming the network of negative-feedback loops. However, the molecular mechanisms of HIF-1α/miR-155–5p link under OTA exposure on ER stress and fibrosis in kidney remain unknown.

The purpose of this study was to demonstrate the underlying mechanism of OTA on hypoxia, ER stress, and renal fibrosis. To the best of our knowledge, this is the first study to evaluate that miR-155–5p is linked to HIFα-1, ER stress, and kidney fibrosis upon OTA exposure.

## 2 Materials and methods

### 2.1 Materials

OTA (CAS no. 303–47–9; >98% purity, benzene-free solid) was obtained from Cfm Oskar Tropitzsch GmbH (Marktredwitz, Germany). Roswell Park Memorial Institute (RPMI) 1640 medium was obtained from Gibco (Grand Island, NY, USA). Fetal bovine serum (FBS), antibiotics (penicillin–streptomycin), trypsin, and ethylenediaminetetraacetic acid were obtained from Hyclone (Logan, UT, USA). Dextrose, sodium bicarbonate, sodium pyruvate, HEPES, siRNA universal negative control (SIC001), and HIF-1α siRNA (SASI HS0200332063) were obtained from Sigma-Aldrich (St. Louis, MO, USA). The miR-155–5p inhibitor (339121) and miRNA inhibitor control (339126) were obtained from Qiagen (Valencia, CA, USA). The miR-155–5p mimic (59089) and miRNA mimic control (0113) were purchased from Genepharma (Shanghai, China). Primary antibodies against HIF-1α (sc-13515), fibronectin (FN, sc-8422), α-smooth muscle actin (α-SMA, sc-53142), activating transcription factor-4 (ATF-4, sc-390063), glucose regulated protein 78 (GRP78, sc-166490) and glyceraldehyde 3-phosphate dehydrogenase (GAPDH, sc-32233) were obtained from Santa Cruz Biotechnology (Dallas, TX, USA) and epithelial cadherin (E-cadherin, 14472 S) was obtained from Cell Signaling (Denver, MA, USA).

### 2.2 Cell culture and treatment

The human proximal tubule epithelial cell (HK-2) was obtained from the Korean Cell Line Bank (Seoul, Republic of Korea). HK-2 cells were cultured in RPMI 1640 medium, containing 2.5 g/L dextrose, 2 g/L sodium bicarbonate, 2.383 g/L 2-[4-(2-hydroxyethyl)− 1-piperazinyl]ethanesulfonic acid (HEPES), 0.11 g/L sodium pyruvate, 100 U/mL antibiotics, and 10% FBS (v/v). The cells were incubated in a humidified incubator at 37 °C conditioned with 5% CO_2_. Dimethyl sulfoxide (DMSO) was used to dissolve OTA to create the working solution (500 µM OTA), which was then diluted with RPMI 1640 medium to establish the final concentrations. The final DMSO concentration was 0.1% in all groups. According to our previous studies, OTA concentrations between 50 and 200 nM induced renal damages and cytotoxicity in kidney cells [27], [28]. Therefore, we applied 25, 50, and 100 nM OTA on HK-2 cells in order to verify the mechanism of toxicity.

### 2.3 Cell transfection

HK-2 cells were transfected with 10 nM miR-155–5p mimic and inhibitor, respectively, for miRNA overexpression and inhibition. For HIF-1 knockdown, HK-2 cells were transfected with 10 nM of HIF-1α siRNA. Transfections were performed using Lipofectamine™ RNAiMAX transfection reagent (Invitrogen, Carlsbad, CA, USA) with Opti-MEM medium (Gibco, Grand Island, NY, USA) for 48 h according to the manufacturer’s protocol. Subsequently, the cells were treated with 100 nM OTA for 48 h.

### 2.4 Animal study

Male ICR mice (6 weeks old, 30–35 g) were obtained from Orientbio, Inc. (Seongnam, Republic of Korea). All experimental procedures were performed in accordance with the guidelines of the Committee for Ethical Usage of Experimental Animals of Korea University (KUIACUC-2021–0026). Mice were kept in an animal experimental facility, at a relative humidity of 50 ± 10%, a constant temperature of 20 ± 3 °C, and 12 h dark/light cycle conditions. Food and water were available to mice without restriction. Before beginning this animal study, the mice were given 7 d for acclimatization, and then randomly selected into three groups (n = 6 per group): (1) Control (CON, 0 mg/kg body weight (bw)), (2) OTA-low (OL, 1 mg/kg bw), (3) OTA-high (OH, 3 mg/kg bw). Based on our previous studies, 1 and 3 mg/kg bw OTA were identified as toxicity inducible concentrations in liver and kidney tissues [27], [29]. Thus, the following experiment applied these concentrations to confirm differentially regulated miRNAs by OTA and its role on renal toxicity. OTA was dissolved in 0.5% NaHCO_3_, and a 200 μL of solution of each concentration was administered by gavage five times per week for 12 weeks.

### 2.5 Histopathological analysis

Kidney tissues (n = 3) were maintained in 10% formalin solution before being embedded in paraffin. The tissues were cut into 4-μm-thick sections using a rotating microtome (Leica Microsystems Ltd., Melbourne, Australia). After deparaffinizing, the sections were stained with Masson’s trichrome and hematoxylin and eosin (H&E) in order to determine the degree of fibrotic and inflammatory damages in kidney, respectively. The sections were visualized with an optical microscope (Olympus, Tokyo, Japan) and a digital slide scanner (VM1, Motic, Beijing, China). The Solution for Automatic Bio-Image Analysis software was used to quantify collagen deposition, which can indicate fibrotic areas (EBIOGEN, Seoul, Republic of Korea).

### 2.6 Blood chemistry analysis

Blood samples were obtained from the ventricular vein of mice and placed in a serum separator tube for 1 h at 25 °C The blood samples were centrifuged for 15 min at 842 × *g* to collect serum. Creatinine level was measured using a blood analyzer (Fuji Dry-Chem3500, Fuji Japan, Tokyo, Japan).

### 2.7 MicroRNA-sequencing (miR-seq)

Total RNA was extracted from kidney tissues using Trizol reagent (Invitrogen, Carlsbad, CA, USA) according to the manufacturer’s protocol. For the quality assessment and quantification of RNA, Agilent 2100 bioanalyzer using the RNA 6000 Pico Chip (Agilent Technologies, Amstelveen, The Netherlands) and NanoDrop 2000 Spectrophotometer system (Thermo Fisher Scientific, Waltham, MA, USA) were used, respectively. The miRNA library was constructed using NEBNext Multiplex SmallRNA Library Prep kit (New England BioLabs, Inc., USA) according to the manufacturer’s protocol.

Sequence reads were mapped in order to obtain bam file by bowtie2 software tool, using mature miRNA sequence as a reference. Read counts mapped on mature miR-seq were extracted from the alignment file using bedtools v2.25.0 [30] and Bioconductor [31] using R statistical programming language [32]. It was also utilized to determine the amount of expression of miRNAs. For comparison between samples, the normalization method of counts per million (CPM) and trimmed mean of M-values (TMM) was used.

### 2.8 Quantitative real-time PCR (qRT-PCR) analysis

Total RNA was extracted from HK-2 cells and kidney tissues using RNAiso Plus (Takara, Kusatu, Japan) after washing with ice-cold phosphate-buffered saline. The extracted RNA of each sample was used to synthesize cDNA using a first-strand cDNA synthesis kit (LeGene Biosciences, San Diego, CA, USA). For miRNA isolation and reverse transcription, miRNeasy Tissue/Cells Advanced Mini Kit (Qiagen, Valencia, CA, USA) and miRCURY LNA RT Kit (Qiagen, Valencia, CA, USA) were used according to the manufacturer`s protocol. Subsequently, EzAMP™ Real-Time qPCR 2X Master Mix SYBR Green (ELPIS Biotech, Daejeon, Korea) was used to measure mRNA expression, and miRCURY LNA SYBR Green PCR kit (Qiagen, Valencia, CA, USA) was used to measure miRNA expression. qRT-PCR was carried out with IQ5 real-time PCR system (Bio-rad, Hercules, CA, USA) following the manufacturer’s instructions. GAPDH and U6 were used as an internal control for the normalization of mRNA and miRNA expression, respectively, and the results were calculated using the 2^-△△CT^ method. All primers used were shown in Supplementary Table 1.

### 2.9 Western blot analysis

HK-2 cells and renal tissues were lysed for 1 h with RIPA lysis buffer (Thermo Fisher Scientific, Waltham, MA, USA) containing 5 μg/mL of aprotinin and leupeptin. After centrifugation at 4 °C and 15,814 x g for 20 min, the supernatant was collected and used as protein lysates. The protein concentrations were examined by BCA protein assay kit (Thermo Fisher Scientific). Subsequently, 40 µg of lysates were separated using 8–10% sodium dodecyl sulfate-polyacrylamide gel electrophoresis, and then transferred to polyvinylidene difuoride membranes (Millipore, Billerica, MA, USA). After blocking using 4–5% skim milk in Tris buffered saline-0.05% (v/v) Tween-20 for 30 min, the membranes were incubated with primary antibodies overnight against HIF-1α (1:500), FN (1:800), α-SMA (1:300), E-cadherin (1:1000), ATF-4 (1:500), GRP78 (1:500), and GAPDH (1:1000) at 4 °C. After that, goat anti-mouse horseradish peroxidase-conjugated secondary antibody (Millipore, Billerica, MA, USA) was used to incubate the membranes at 25 °C for 1 h, and protein bands were detected with ECL™ Select western blotting detection reagent (Cytiva, Buckinghamshire, UK). The images were analyzed with ImageQuant™ LAS 400 mini (GE Healthcare, Buckinghamshire, UK), which were then quantified by ImageJ analysis (National Institutes of Health, Bethesda, MD, USA). GAPDH was used as an internal control.

### 2.10 Statistical analysis

All data are presented as the mean ± standard deviation (SD). All statistical analyses were conducted using SAS version 9.4 (SAS Institute, Cary, NC, USA). Duncan’s multiple range test was performed for in vivo experiments, and Tukey’s multiple range test or student’s *t* test were performed for in vitro experiments. Differences with *p* < 0.05 were considered as statistically significant.

## 3 Results

### 3.1 OTA increased serum creatinine levels and caused histopathological changes in kidney tissues

**Fig. 1:**
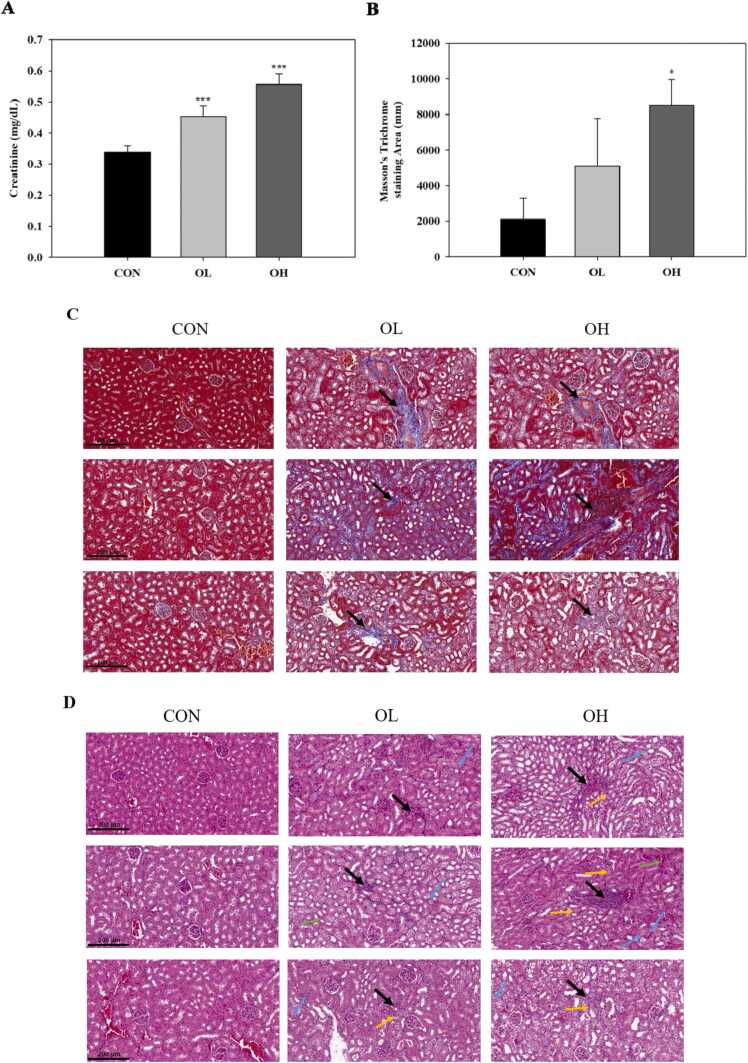
Ochratoxin A (OTA) increased serum creatinine levels and caused histopathological changes in kidney tissues. A: Creatinine levels. B: Masson’s trichrome-stain section area of kidney tissues. C: Kidney tissues examined by Masson’s trichrome staining. Black arrows: fibrosis location. D: Kidney tissues examined by hematoxylin and eosin staining. Black arrows: interstitial nephritis, green arrows: loss of brush border, blue arrows: swelling in tubular epithelial cell, yellow arrows: apoptosis. Significant differences between control and test groups were expressed using Duncan’s multiple range test. * *p* < 0.05, and *** *p* < 0.001. CON, the control group treated with vehicle only; OL, the group treated with OTA 1 mg/kg body weight (bw); OH, the group treated with OTA 3 mg/kg bw.

To examine the negative effects of OTA on renal dysfunction, an in vivo model was established by orally administering OTA into mice. The blood creatinine level in mice exposed to OTA was found to be significantly (*p* < 0.001) greater than that in control mice (Fig. 1 A). Masson’s trichrome staining was carried out to observe positive areas of extracellular matrix, exhibited as blue stained areas around the interstitial space, which could indicate the degree of fibrosis. As shown in Fig. 1 B-C, the stained regions increased significantly (*p* < 0.05) in the OTA (3 mg/kg bw) administered group (OH group) as compared to the control group and the OTA (1 mg/kg bw) given group (OL group). The similar fibrotic change was seen in the OL group, although it was not statistically significant when compared to the control group. Meanwhile, H&E staining revealed the presence of interstitial nephritis (black arrow), brush border loss (green arrow), swelling in tubular epithelial cells (blue arrow), and apoptosis (yellow arrow) in OTA-treated groups (Fig. 1 D). These findings revealed that OTA produced fibrotic and inflammatory damage in kidney tissues.

### 3.2 OTA induced hypoxia-like conditions such as ER stress and fibrosis in vitro and in vivo

**Fig. 2:**
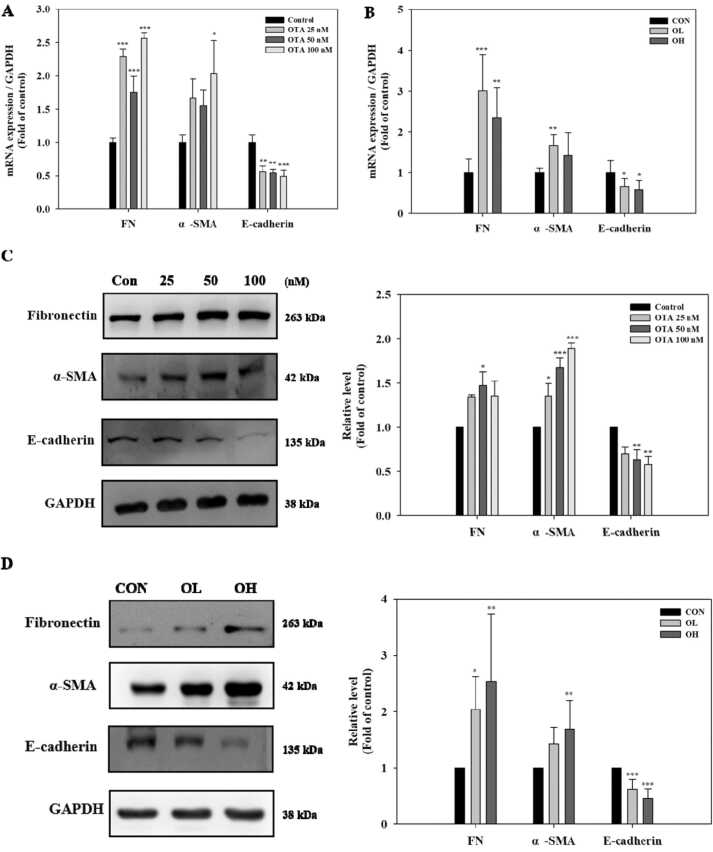

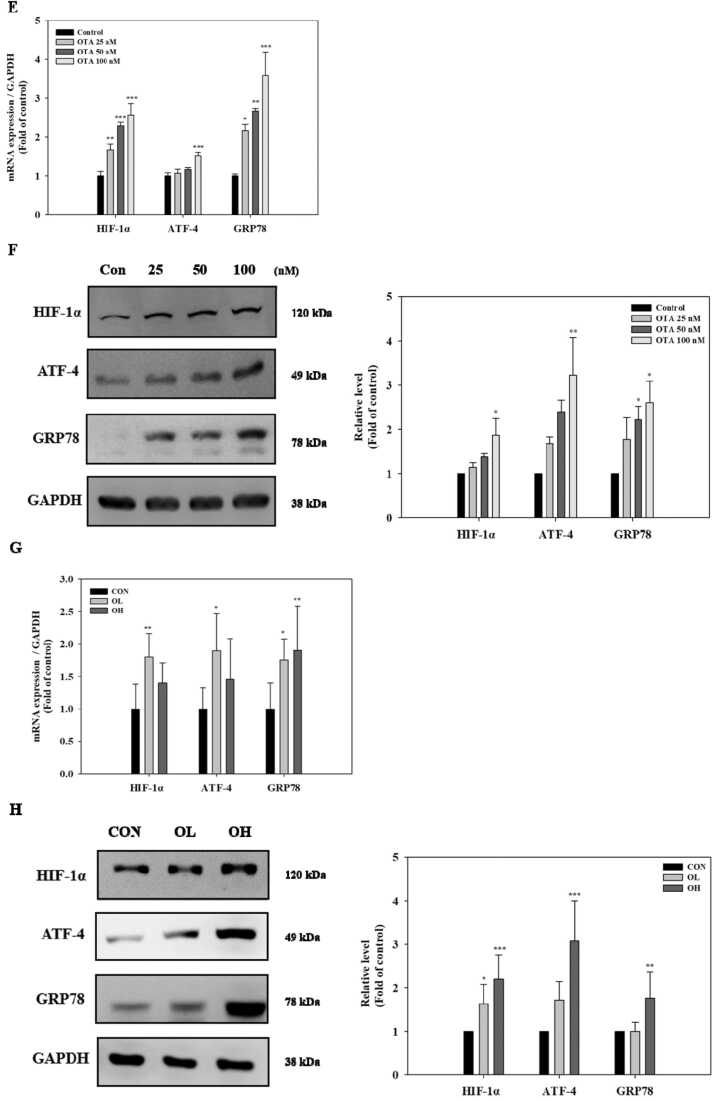
OTA induced hypoxia-like conditions such as endoplasmic reticulum (ER) stress and fibrosis in HK-2 cells and kidney tissues. The mRNA levels of A/C in HK-2 and B/D in kidney tissues, and the protein expression levels of E/F in HK-2 cells and G/H in kidney tissues were shown. Regarding in HK-2 cells and in kidney tissues results, significant differences between control and test groups were expressed using Tukey’s multiple range test and Duncan’s multiple range test, respectively. * *p* < 0.05, ** *p* < 0.01, and *** *p* < 0.001. CON, the control group treated with vehicle only; OL, the group treated with OTA 1 mg/kg bw; OH, the group treated with OTA 3 mg/kg bw.

To evaluate whether OTA treatment modulates hypoxia, ER stress, and fibrosis, HK-2 cells, which were treated with OTA (25, 50, and 100 nM), and kidney tissues, which were given OTA (1 and 3 mg/kg bw) were analyzed. In the in vitro experiment, FN mRNA expression level was significantly (*p* < 0.001) higher in OTA-treated cells than in control cells, while α-SMA mRNA expression level was significantly (*p* < 0.05) higher in 100 nM OTA treated cells (Fig. 2A). Similarly, the FN mRNA expression level was significantly (at least *p* < 0.01) elevated in kidney tissues in the OTA-fed groups, while α-SMA mRNA expression level was significantly (*p* < 0.01) higher in the OL group (Fig. 2 B). Significant (at least *p* < 0.05) increases in protein expression levels of FN and α-SMA were found in OTA-treated cells at 50 nM OTA and all tested OTA levels, respectively. However, when compared to control cells and animal group, the mRNA and protein levels of E-cadherin, an epithelial marker, were significantly (at least *p* < 0.05) downregulated in cells (Fig. 2A and C) and kidney tissues with OTA treatment (in the case of protein levels in cells, only those treated with 50 and 100 nM OTA) (Fig. 2 B and D). For the hypoxia and ER stress markers, the mRNA and protein expression levels of HIF-1α, a crucial factor regulating the response for adaptation to hypoxia, and ER stress responsive proteins including ATF-4 and GRP78 were measured. *In vitro*, the expressions of mRNA HIF-1α and GRP78 were significantly (at least *p* < 0.05) higher in the OTA-treated cells than in the control cells. In cells treated with 100 nM OTA, there was a substantial (*p* < 0.001) increase in ATF-4 mRNA expression (Fig. 2 E). HIF-1α and ATF4 protein expression levels were significantly (at least *p* < 0.05) greater after 100 nM OTA treatment, while GPR78 protein expression levels were significantly (*p* < 0.05) higher after 50 nM OTA treatment (Fig. 2 F). *In vivo*, HIF-1α and ATF-4 mRNA levels were significantly (at least *p* < 0.05) higher in the OL group, whereas GPR78 mRNA levels were significantly (at least *p* < 0.05) higher in both the OL and OH groups (Fig. 2G). HIF-1α protein levels increased significantly (at least p < 0.05) in the OL and OH groups, respectively, whereas ATF-4 and GRP78 protein levels increased significantly (at least *p* < 0.01) in the OH group (Fig. 2 H).

### 3.3 Identification of differentially expressed miRNAs in kidney tissue of OTA-treated mice

**Fig. 3:**
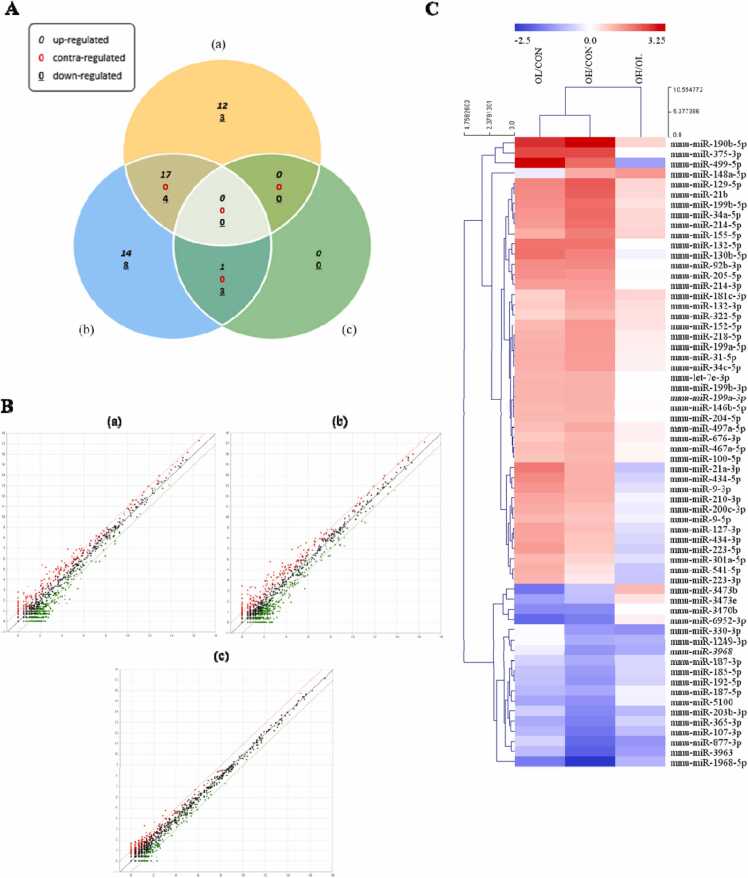
Identification of differentially expressed miRNAs (DEMs) in kidney tissues of OTA-treated mice. Kidney tissues were analyzed using microRNA-sequencing (miR-seq) in order to determine differentially expressed miRNAs in the presence of OTA. Each comparison means DEMs in the OL group compared with CON group ((a) OL/CON), the OH group compared with CON group ((b), OH/CON), and OL group compared with OH group ((c), OH/OL), respectively. Pairwise comparisons among the CON, OL, and OH groups are shown in A: Venn diagram, showing the DEMs and overlap, B: Scatter plots, and C: Heatmap. CON, the control group treated with vehicle only; OL, the group treated with OTA 1 mg/kg bw; OH, the group treated with OTA 3 mg/kg bw.

To profile miRNA expression using miR-seq, kidney tissues were collected from 9 mice, including the control (n = 3), OL (n = 3) and OH group (n = 3). Differentially expressed miRNAs (DEMs) were visualized in the pairwise comparisons among the control, OL, and OH groups, presented as venn diagram, scatter plots, and heatmaps (Fig. 3A to C). OTA exposure induced the differential expression in total 62 miRNAs, which had significant (*p* < 0.05) differential expression of > 2.0-fold change. The OL and OH group displayed 36 DEMs (29 upregulated, 7 downregulated) and 47 DEMs (32 upregulated, 15 downregulated), compared to the control group, respectively. Furthermore, compared to the OL group, 4 DEMs (1 upregulated, 3 downregulated) were found in the OH group (Fig. 3A). Information on the top 62 miRNAs in detail was listed in Fig. 3C and Supplementary Table 2.

**Fig. 4:**
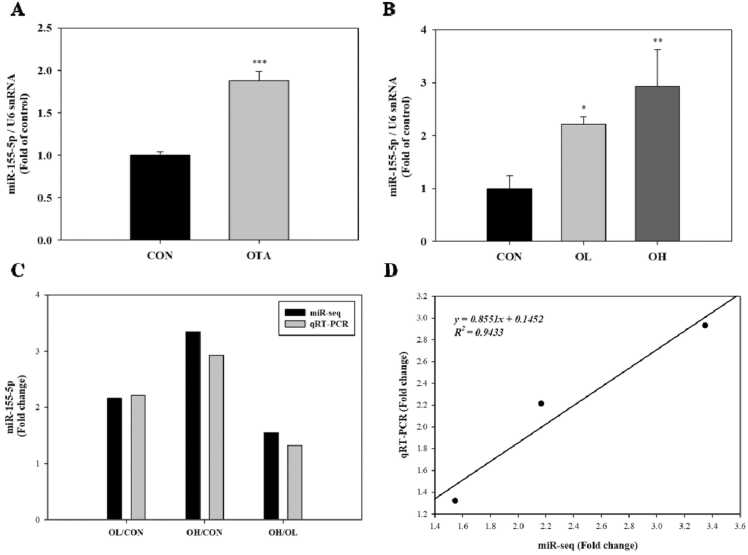
Validation of the micro RNA-155–5p (miR-155–5p) expression in kidney after OTA exposure. A: miR-155–5p levels measured using qRT-PCR in OTA treated-HK-2 cells. B: miR-155–5p levels in kidney tissues after OTA exposure. Cells were treated with 100 nM OTA for 48 h. C: The results of miR-155–5p levels from miR-seq and qRT-PCR were determined by pairwise comparison among the control, OL, and OH group. D**:** Correlation plot between fold change of miR-seq and qRT-PCR. Significant differences between control and test groups were expressed using by Student’s t-test for in vitro and Duncan’s multiple range test for in vivo experiment. * *p* < 0.05, ** *p* < 0.01, and *** *p* < 0.001. CON, the control group treated with vehicle only; OL, the group treated with OTA 1 mg/kg bw; OH, the group treated with OTA 3 mg/kg bw.

According to previous research, OTA is implicated in the processes that drive protein processing in the ER, and hypoxia, EMT, and UPR may be important OTA-induced effects in kidney epithelial cells [27]. We focused on miR-155–5p among the 62 DEMs since it has been linked to hypoxia, ER stress, and fibrosis [24], [25]. MiR-155–5p has been linked to ER-stress-induced cardiomyocyte apoptosis and renal interstitial fibrosis in obstructive nephropathy, according to their research. In the present study, there was a remarkable induction of miR-155–5p in the kidney tissues of OTA-treated groups with the higher reliability (Fig. 4).

### 3.4 Validation of the miR-155–5p expression in kidney after OTA exposure

To validate the miR-seq results of miR-155–5p, we performed qRT-PCR on HK-2 cells and kidney tissues. As with the miR-seq data, qRT-PCR results of miR-155–5p expression indicated a consistent pattern to increase in the 100 nM OTA-treated cells and the OL and OH groups, with similar extents of fold change (Fig. 4A to C). MiR-155–5p expression increased significantly in the OTA-treated cells (*p* < 0.001), as well as in the OL (fold change=2.215; *p* < 0.05) and OH groups (fold change=2.932; *p* < 0.01)) as compared to the respective control (Fig. 4A and B). Furthermore, compared to the OL group, there was a higher level of miR-155–5p in OH group as well (fold change=1.324) (Fig. 4B). Correlation analysis employing fold changes among the control, OL, and OH groups revealed an R^2^ of 0.9433, confirming high linearity between miR-seq and qRT-PCR data (X = fold changes from miR-seq, Y=fold changes from qRT-PCR) (Fig. 4 D).

### 3.5 Knockdown of HIF-1α downregulated miR-155–5p and alleviated ER stress and fibrosis in OTA-treated HK-2 cells

**Fig. 5:**
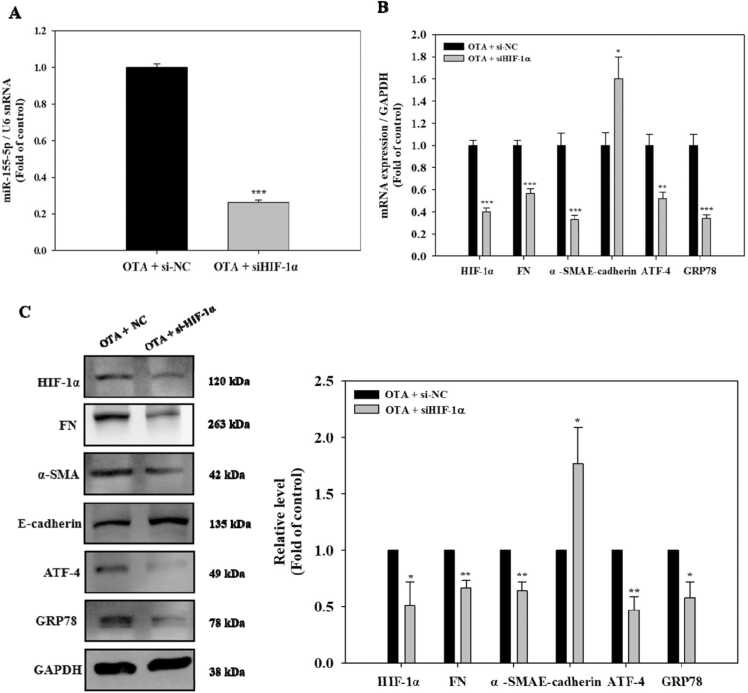
Knockdown of HIF-1α downregulated miR-155–5p and alleviated ER stress and fibrosis in OTA-exposed HK-2 cells. Cells were transfected using siRNA control (si-NC) or siHIF-1α followed by exposure of 100 nM OTA for 48 h. A: miR-155–5p levels. B: mRNA levels of HIF-1α, fibrosis, and ER stress markers. C: protein levels of HIF-1α, fibrosis, and ER stress markers. Significant difference between control and the test group was expressed using Student’s t-test, * *p* < 0.05, ** *p* < 0.01, and *** *p* < 0.001.

HK-2 cells were exposed to 100 nM OTA after HIF-1α was knocked down to determine if HIF-1α is involved in the induction of miR-155–5p and the development of renal ER stress and fibrosis in the presence of OTA. OTA-induced expression of miR-155–5p was significantly (*p* < 0.001) reduced following HIF-1 knockdown, as seen in Fig. 5A. The mRNA and protein expressions of HIF-1α significantly (at least *p* < 0.05) decreased with HIF-1α si RNA (siHIF-1α) transfection, compared to the siRNA negative control (si-NC) transfection in the cells treated with OTA (Fig. 5B and C). Following that, we examined the levels of ER stress and fibrosis markers in HIF-1-silenced HK-2 cells exposed to OTA. In compared to control cells, the mRNA and protein levels of FN, α-SMA, ATF-4, and GRP78 were significantly (at least *p* < 0.05) lowered in the HIF-1-silenced cells exposed to OTA (Fig. 5B and C). In contrast, HIF-1α silencing significantly (*p* < 0.05) upregulated the E-cadherin expressions. These findings showed that HIF-1α had a significant role in the OTA-induced expression of miR-155-p and genes linked to ER stress and fibrosis in kidney epithelial cells.

### 3.6 Inhibition of miR-155–5p attenuated ER stress and fibrosis in OTA-exposed HK-2 cells

**Fig. 6:**
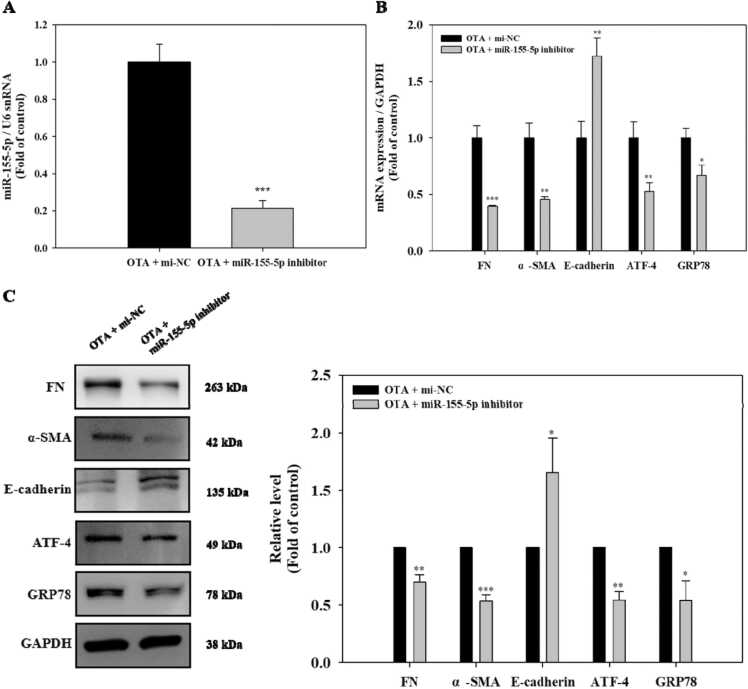
Inhibition of miR-155–5p attenuated ER stress and fibrosis in OTA-exposed HK-2 cells. Cells were transfected using miRNA control (mi-NC) or miR-155–5p inhibitor followed by exposure of OTA (100 nM) for 48 h. A: miR-155–5p levels. B: mRNA levels of ER stress and fibrosis markers. C: protein levels of fibrosis and ER stress markers. Significant difference between control and the test group was expressed using Student’s t-test, * *p* < 0.05, ** *p* < 0.01, and *** *p* < 0.001.

The HK-2 cells were transfected with the miR-155–5p inhibitor, and then the cells were treated with 100 nM OTA to determine if miR-155–5p was the source of the ER stress and fibrosis generated by OTA in the kidney. Comparing the miR-155–5p expression in the inhibitor-transfected cells to the miRNA negative control (mi-NC)-transfected cells, there was a significantly (*p* < 0.001) reduced expression (Fig. 6A). Following that, the levels of ER stress and fibrosis markers in miR-155–5p-inhibited HK-2 cells exposed to OTA were measured. As demonstrated in Fig. 6B and C, there were significantly (at least *p* < 0.05) decreased mRNA and protein levels of FN, α-SMA, ATF-4, and GRP78 in miR-155–5p-inhibited cells. In contrast, cells with miR-155–5p inhibition had significantly (at least *p* < 0.05) higher E-cadherin mRNA and protein levels than control cells. Thus, our study demonstrated that miR-155–5p plays a role in increasing ER stress and fibrosis in OTA-treated HK-2 cells.

### 3.7 HIF-1α induced ER stress and fibrosis in OTA-exposed HK-2 cells via upregulating miR-155–5p

**Fig. 7:**
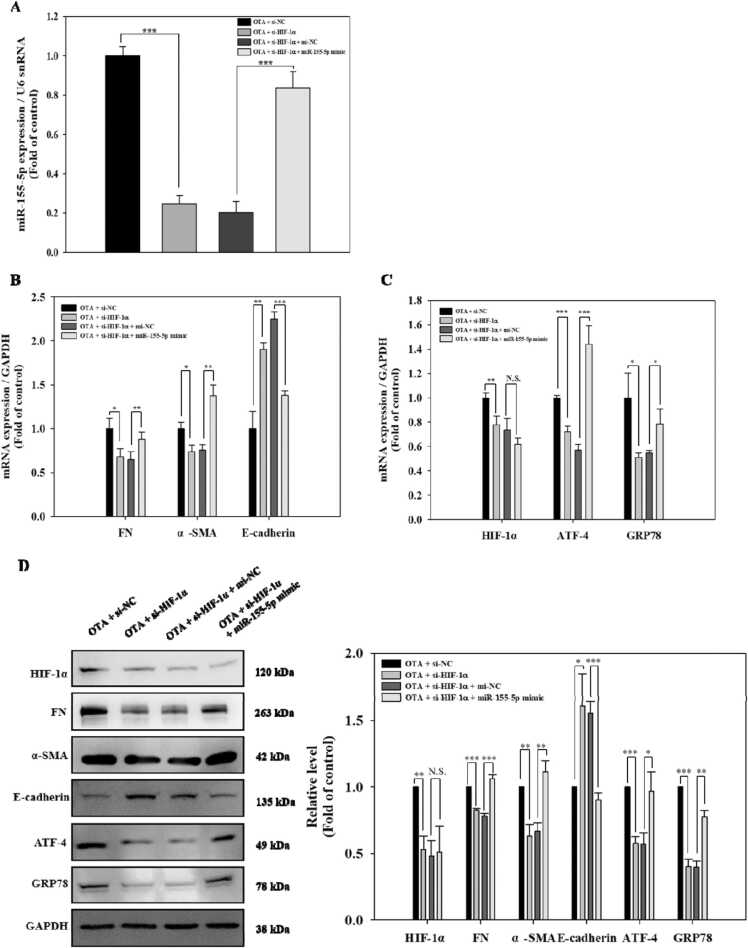
HIF-1α induced ER stress and fibrosis in OTA-exposed HK-2 cells via upregulating miR-155–5p. Cells were transfected using si-NC or si-HIF-1α, and mi-NC or miR-155–5p mimic followed by exposure of 100 nM OTA for 48 h. A: miR-155–5p levels B-C: mRNA expression of HIF-1α, fibrosis, and ER stress markers. D: protein levels of HIF-1α, fibrosis, and ER stress markers. Significant differences were expressed using Student’s t-test, * *p* < 0.05, ** *p* < 0.01, and *** *p* < 0.001 (OTA + si-NC vs OTA + si-HIF-1α, and OTA + si-HIF-1α + mi-NC vs OTA + si-HIF-1α + miR-155–5p mimics).

To examine if HIF-1α-induced ER stress and fibrosis is dependent on miR-155–5p during OTA exposure, HK-2 cells were co-transfected with siHIF-1α and miR-155–5p mimic before being exposed to 100 nM OTA. MiR-155–5p expression was significantly (*p* < 0.001) suppressed following HIF-1α silencing, whereas miR-155–5p overexpression significantly (*p* < 0.001) restored this decrease in response to OTA exposure (Fig. 7A). Whereas HIF-1α-silenced cells demonstrated downregulation of FN, α-SMA, ATF-4, and GRP78 and upregulation of E-cadherin in the presence of OTA compared to the si-NC transfected cells, these phenomena were significantly (at least *p* < 0.05) reversed following miR-155–5p overexpression Fig. 7B and C). Meanwhile, HIF-1α knockdown effectively inhibited the level of HIF-1α in OTA-induced HK-2 cells, while miR-155–5p overexpression had little influence on HIF-1α expression (Fig. 7C and D). These data suggested that OTA promoted ER stress and fibrosis through HIF-1α-linked miR-155–5p signaling pathway in HK-2 cells.

## 4 Discussions

OTA is a common fungal toxin that is odorless, white, crystalline, and thermally stable, with limited water solubility [33], [34]. Several organs are affected by its toxicity, but the kidney is the most severely affected [35]. For instance, chronic ingestion of OTA in food may contribute to the development of endemic nephropathy [36]. Several findings revealed OTA as a potent renal carcinogen in animals [37]. However, studies on the mechanism of action of OTA on renal damage are still limited. Recent studies have shown that a number of miRNAs play important roles in UPR signaling, ER homeostasis, and the onset of renal fibrosis [38], [39]. We attempted to understand whether there is a link between HIF-1α, the key regulator of hypoxia, and miR-155–5p in OTA-induced renal ER stress and fibrosis.

Renal hypoxia, characterized by decreased oxygen tension in the kidney, and ER stress are prognostic markers for CKD progression [40]. HIF-1α, the major transcription factor regulating tissue response to low oxygen, modulates cellular adaptation to hypoxia [41]. According to recent studies, hypoxia causes an ER stress response, which correlates with increased cancer cell proliferation [42]. The protein folding process may be altered under hypoxia, promoting the accumulation of misfolded/unfolded proteins and impairing ER homeostasis, which results in UPR activation [43]. The UPR causes the dissociation of GRP78, a master regulator of ER stress sensors, from transmembrane sensors in the ER, activating ER-stress related markers such as PERK/ATF-4, IRE1, and ATF6 [44]. In the context of CKD, activation of these markers might lead to inflammation, cell death, and fibrosis [45], [46]. Renal fibrosis is the final stage of CKD, characterized by widespread deposition and accumulation of ECM components such as fibronectin, which eventually leads to renal failure [47], [48]. EMT is one of the main driving forces in the development of fibrosis, characterized as increased EMT indicators, such as α-SMA, while adhesion molecules of epithelial cells, such as E-cadherin decreased [49]. In recent studies, HIF-1α caused liver fibrosis via PTEN/p65 signaling and interstitial fibrosis via modulation of EMT [50], [51]. We showed that OTA produced hypoxia-like conditions such as ER stress, and fibrosis by increasing the expression of HIF-1α, ER stress associated proteins (ATF-4 and GRP78), and fibrotic markers (FN and α-SMA) while decreasing the expression of E-cadherin. Furthermore, the regulatory effect of HIF-1α on OTA-induced ER stress and fibrosis was demonstrated: siRNA-mediated HIF-1α silencing inhibited the upregulation of FN, -SMA, ATF-4, GRP78, and downregulation of E-cadherin during OTA exposure. These findings suggest that HIF-1α may be a novel regulator of OTA-induced ER stress and fibrosis in the kidney.

MiRNAs have various regulatory functions in renal physiology, including renal homeostasis, development, and vascular calcification [20]. Numerous renal disorders have been linked to miRNA dysregulation, suggesting that miRNAs may serve as therapeutic biomarkers for kidney damage [52]. For instance, the miR-200b inhibitor recovered high glucose-caused ER stress, as well as UPR signaling, through regulation of CITED2 [38]. It was found that miR-27b-3p overexpression alleviated renal fibrosis in TGF-β1-induced HK-2 cells through STAT1 inactivation [53]. We analyzed DEMs with renal tissues using next generation sequencing (NGS) to determine the miRNAs regulated by OTA. Among the 62 miRNAs that were differently expressed (p < 0.05, Fold change > 2.0) in response to OTA, miR-155–5p has been shown to promote liver damage via oxidative stress-mediated ER stress and function as a biomarker for hepatic fibrosis [54], [55]. Increased miR-155 was found in an independent patient cohort in unbiased profiling approaches to discover a urine miRNA signature linked with diabetic kidney disease [56]. Furthermore, miR-155, which was shown to be overexpressed in acute rejection biopsies, was found to be strongly expressed in peripheral blood mononuclear cells [57]. Given that HIF-1α may stimulate miR-155–5p in colon cancer cells [26], we hypothesized that the possible link of HIF-1α/miR-155–5p could accelerate OTA-induced ER stress and fibrosis in kidney.

In the current research, we revealed that silencing of HIF-1α considerably blocked miR-155–5p induction in OTA-exposed HK-2 cells. The upregulation of ATF-4, GRP78, FN and α-SMA and downregulation of E-cadherin after being treated with OTA were suppressed when HIF-1α and miR-155–5p were silenced, respectively. Furthermore, the restrained expressions of ER stress and fibrotic markers by HIF-1α knockdown were recovered by miR-155–5p mimic. It should be noted that the mimic of miR-155–5p had no impact on the expression of HIF-1α. The regulatory mechanism of miRNAs by HIF-1α was reviewed by Peng et al. [58]. According to the review, HIF-1α directly regulates noncoding RNAs (ncRNAs), including miRNAs, at the transcriptional level via hypoxia-responsive elements found in the promoter regions of ncRNAs, and also indirectly regulates them via epigenetic processes mediated by histone deacetylases. MiR-210 mediated HIF-1α-induced EMT to drive invasion of gastric cancer by regulating homeobox 49 expression [59]. During normoxia, the proline hydroxylases-pVHL-proteasome system rapidly degrades HIF-1α; however, during hypoxia, HIF-1α is stabilized and translocated into the nucleus, where it dimerizes with aryl hydrocarbon receptor nuclear translocator (ARNT), also designated as HIF-1β and forms a transcriptionally active HIF complex [60]. Supplementary Fig. 1 clearly illustrates that treatment of HK-2 cells with 50 nM OTA significantly (*p* < 0.05) increased the protein expression levels of HIF-1α and ARNT in the nucleus. Hypoxic conditions are also important for the production of argonaute proteins. HIF-1α, in particular, increases the development of the protein argonaute-4 [EIF2C4], which is known to induce mir-155 overexpression [61]. According to the research, there are a limited number of hypoxia-induced miRNAs, and the majority of them may alter HIF-related miRNAs [62]. To determine if the promoter region of miR-155–5p contains any consensus HIF1-α binding elements, localization studies in either the mouse or HK2 experiments should be done. We may postulate that HIF-1α increased ER stress and fibrosis in HK-2 cells via upregulating miR-155–5p. Furthermore, additional research surely remains to understand the various roles of other OTA-modulated miRNAs on expression of target genes in OTA-induced kidney injury.

To our knowledge, this study figured out for the first time the OTA-induced HIF-1α–miR-155–5p circuit as a pivotal regulatory pathway for renal ER stress and fibrosis. We, therefore, have revealed a potential link between OTA-induced renal ER stress/fibrosis and HIF-1α–miR-155–5p circuit, which might have a potential role in OTA-induced chronic nephropathy.

## CRediT authorship contribution statement

**Seon Ah Yang:** Data curation, Writing – original draft. **Kyu Hyun Rhee:** Methodology. **Hee Joon Yoo:** Visualization, Investigation. **Min Cheol Pyo:** Conceptualization. **Kwang-Won Lee:** Supervision, Writing – review & editing.

## Declaration of Competing Interest

The authors declare that they have no known competing financial interests or personal relationships that could have appeared to influence the work reported in this paper.

## Data Availability

Data will be made available on request. The data that has been used is confidential.
