# Supplementary material for: Ochratoxin A induces endoplasmic reticulum stress and fibrosis in the kidney via the HIF-1α/miR-155-5p link
Source: Toxicol Rep. 2023 Jan 18;10:133–45. doi: 10.1016/j.toxrep.2023.01.006 (PMC9879730; doi:10.1016/j.toxrep.2023.01.006)
Supplement: Supplementary file 1 — Supplementary material [file mmc1.docx]

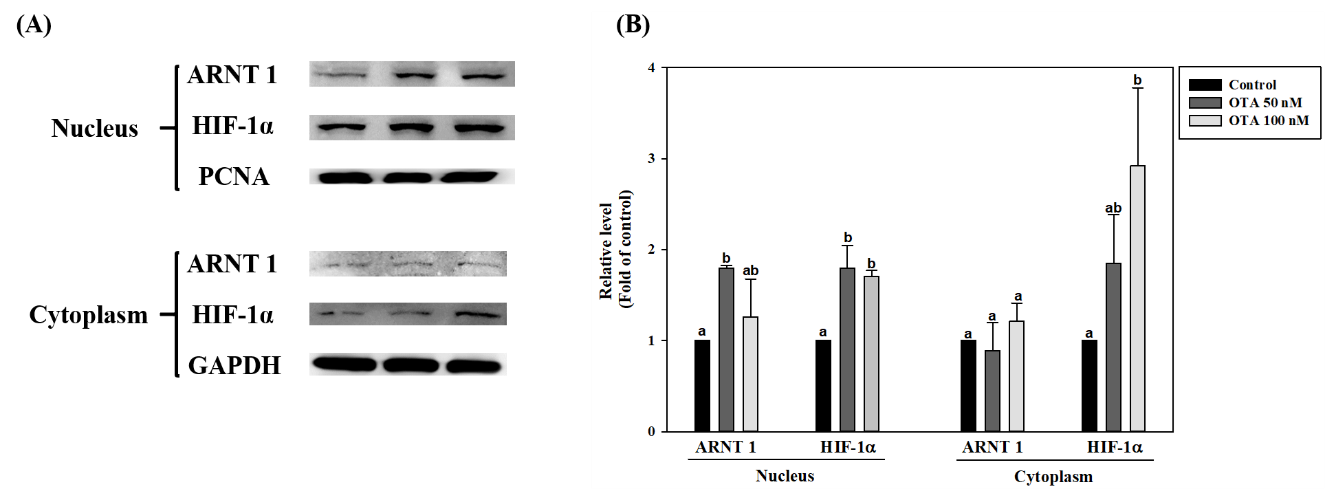


**Supplementary figure S1. Ochratoxin A (OTA) increased nucleus protein expression levels of HIF-1α and ARNT 1 in HK-2 cells.** The HK-2 cells were treated OTA 0, 50, and 100 nM for 48 h. The assay was performed by qRT-PCR. The protein expression level of ARNT 1 and HIF-1α was expressed by the fold of control. Significant differences between control and test groups were expressed using Tukey’s multiple range test and the different letters indicate the significance.
