# Supplementary material for: Ochratoxin A induces endoplasmic reticulum stress and fibrosis in the kidney via the HIF-1α/miR-155-5p link
Source: Toxicol Rep. 2023 Jan 18;10:133–45. doi: 10.1016/j.toxrep.2023.01.006 (PMC9879730; doi:10.1016/j.toxrep.2023.01.006)
Supplement: Supplementary file 2 — Supplementary material [file mmc2.docx]

**Supplementary table 1.** qRT-PCR primer sequences

| Origin | Marker | Sequence (5’🡪3’) | |
| --- | --- | --- | --- |
| Human | Fibronectin | Forward Reverse | CTG GCC GAA AAT ACA TTG TAA A CCA CAG TCG GGT CAG GAG |
|  | α-SMA | Forward Reverse | CTC TCT GTC CAC CTT CCA G TAA CGA GTC AGA GCT TTC GC |
|  | E-cadherin | Forward Reverse | GCC TCC TGA AAA GAG AGT GGA AG TGG CAG TGT CTC TCC AAA TCC G |
|  | ATF-4 | Forward Reverse | CCA ACA ACA GCA AGG AGG AT GGG GCA AAG AGA TCA CAA GT |
|  | GRP78 | Forward Reverse | AGT GGT GCC TAC CAA GAA GTC TCA TGT CAG GGG TCT TTC ACC TTC ATA |
|  | GAPDH | Forward Reverse | TGC ACC ACC AAC TGC TTA GC GGC ATG GAC TGT GGT CAT GAG |
| Mouse | Fibronectin | Forward Reverse | CAC GAT GCG GGT CAC TTG CTG CAA CGT CCT CAT TCT TC |
|  | α-SMA | Forward Reverse | TCC TGA CGC TGA ACT ATC CG GGC CAC ACG AAG CTC CTT AT |
|  | E-cadherin | Forward Reverse | CCA AGC AGC AGT ACA TTC TAC A CAT TCA CAT CCA GCA CAT CCA |
|  | ATF-4 | Forward Reverse | GAG CTT CCT GAA CAG CGA AGT G TGG CCA CCT CCA GAT AGT CAT C |
|  | GRP78 | Forward Reverse | GAA AGG ATG GTT AAT GAT GCT GAG GTC TTC AAT GTC CGC ATC CTG |
|  | GAPDH | Forward Reverse | AGA ACA TCA TCA TCC CTG CAT CCA CCG TTC AGC TCT GGG ATC AC |

**Supplementary table 2.** Differentially expressed miRNAs in renal tissues of OTA-treated mice

| Mature miRNAs | Pairwise comparison | Fold change | Mature miRNAs | Pairwise comparison | Fold change |
| --- | --- | --- | --- | --- | --- |
| Up-regulated miRNAs | | | Down-regulated miRNAs | | |
| miR-190b-5p | OL/CON | 6.242 | miR-1968-5p | OL/CON | 0.326 |
|  | OH/CON | 9.487 |  | OH/CON | 0.174 |
| miR-375-3p | OL/CON | 5.205 | miR-3963 | OL/CON | 0.574 |
|  | OH/CON | 5.244 |  | OH/CON | 0.254 |
| miR-129-5p | OL/CON | 3.118 | miR-877-3p | OL/CON | 0.686 |
|  | OH/CON | 4.624 |  | OH/CON | 0.283 |
| miR-21b | OL/CON | 3 | miR-107-3p | OL/CON | 0.551 |
|  | OH/CON | 4.37 |  | OH/CON | 0.313 |
| miR-34a-5p | OL/CON | 2.736 | miR-6952-3p | OL/CON | 0.28 |
|  | OH/CON | 4.008 |  | OH/CON | 0.334 |
| miR-499-5p | OL/CON | 8.961 | miR-365-3p | OL/CON | 0.491 |
|  | OH/CON | 3.988 |  | OH/CON | 0.342 |
| miR-199b-5p | OL/CON | 2.915 | miR-203b-3p | OL/CON | 0.668 |
|  | OH/CON | 3.923 |  | OH/CON | 0.37 |
| miR-214-5p | OL/CON | 2.596 | miR-3470b | OL/CON | 0.374 |
|  | OH/CON | 3.813 |  | OH/CON | 0.374 |
| miR-132-5p | OL/CON | 3.68 | miR-5100 | OL/CON | 0.471 |
|  | OH/CON | 3.69 |  | OH/CON | 0.399 |
| miR-155-5p | OL/CON | 2.166 | miR-3968 | OL/CON | 0.842 |
|  | OH/CON | 3.347 |  | OH/CON | 0.41 |
| miR-130b-5p | OL/CON | 3.745 | miR-192-5p | OL/CON | 0.595 |
|  | OH/CON | 3.227 |  | OH/CON | 0.412 |
| miR-92b-3p | OL/CON | 2.943 | miR-330-3p | OL/CON | 1.062 |
|  | OH/CON | 3.062 |  | OH/CON | 0.422 |
| miR-205-5p | OL/CON | 2.823 | miR-185-5p | OL/CON | 0.609 |
|  | OH/CON | 2.718 |  | OH/CON | 0.432 |
| miR-152-5p | OL/CON | 1.951 | miR-187-5p | OL/CON | 0.552 |
|  | OH/CON | 2.635 |  | OH/CON | 0.481 |
| miR-218-5p | OL/CON | 2.096 | miR-187-3p | OL/CON | 0.692 |
|  | OH/CON | 2.571 |  | OH/CON | 0.491 |
| miR-199a-5p | OL/CON | 2.12 | miR-1249-3p | OL/CON | 0.957 |
|  | OH/CON | 2.561 |  | OH/CON | 0.496 |
| miR-31-5p | OL/CON | 2.141 | miR-3473e | OL/CON | 0.447 |
|  | OH/CON | 2.538 |  | OH/CON | 0.584 |
| miR-34c-5p | OL/CON | 2.136 | miR-3473b | OL/CON | 0.309 |
|  | OH/CON | 2.518 |  | OH/CON | 0.599 |
| miR-214-3p | OL/CON | 2.549 | - | - | - |
|  | OH/CON | 2.488 |  | - | - |
| miR-181c-3p | OL/CON | 1.564 | - | - | - |
|  | OH/CON | 2.414 |  | - | - |
| miR-132-3p | OL/CON | 1.66 | - | - | - |
|  | OH/CON | 2.278 |  | - | - |
| miR-497a-5p | OL/CON | 1.886 | - | - | - |
|  | OH/CON | 2.266 |  | - | - |
| miR-148a-5p | OL/CON | 0.838 | - | - | - |
|  | OH/CON | 2.206 |  | - | - |
| miR-21a-3p | OL/CON | 3.501 | - | - | - |
|  | OH/CON | 2.132 |  | - | - |
| miR-434-5p | OL/CON | 3.061 | - | - | - |
|  | OH/CON | 2.126 |  | - | - |
| miR-9-3p | OL/CON | 2.747 | - | - | - |
|  | OH/CON | 2.119 |  | - | - |
| let-7e-3p | OL/CON | 2.042 | - | - | - |
|  | OH/CON | 2.117 |  | - | - |
| miR-199b-3p | OL/CON | 2.065 | - | - | - |
|  | OH/CON | 2.116 |  | - | - |
| miR-146b-5p | OL/CON | 1.985 | - | - | - |
|  | OH/CON | 2.114 |  | - | - |
| miR-199a-3p | OL/CON | 2.1 | - | - | - |
|  | OH/CON | 2.107 |  | - | - |
| miR-467a-5p | OL/CON | 1.856 | - | - | - |
|  | OH/CON | 2.091 |  | - | - |
| miR-210-3p | OL/CON | 2.338 | - | - | - |
|  | OH/CON | 2.054 |  | - | - |
| miR-322-5p | OL/CON | 1.527 | - | - | - |
|  | OH/CON | 2.045 |  | - | - |
| miR-204-5p | OL/CON | 1.958 | - | - | - |
|  | OH/CON | 2.041 |  | - | - |
| miR-676-3p | OL/CON | 1.695 | - | - | - |
|  | OH/CON | 2.032 |  | - | - |
| miR-100-5p | OL/CON | 1.778 |  |  |  |
|  | OH/CON | 2.009 |  |  |  |
| miR-127-3p | OL/CON | 2.427 |  |  |  |
|  | OH/CON | 1.849 |  |  |  |
| miR-200c-3p | OL/CON | 2.223 |  |  |  |
|  | OH/CON | 1.845 |  |  |  |
| miR-9-5p | OL/CON | 2.025 |  |  |  |
|  | OH/CON | 1.751 |  |  |  |
| miR-434-3p | OL/CON | 2.396 |  |  |  |
|  | OH/CON | 1.74 |  |  |  |
| miR-223-5p | OL/CON | 2.555 |  |  |  |
|  | OH/CON | 1.726 |  |  |  |
| miR-301a-5p | OL/CON | 2.01 |  |  |  |
|  | OH/CON | 1.535 |  |  |  |
| miR-541-5p | OL/CON | 2.173 |  |  |  |
|  | OH/CON | 1.354 |  |  |  |
| miR-223-3p | OL/CON | 2.048 |  |  |  |
|  | OH/CON | 1.267 |  |  |  |

**miRNA**, microRNA;

**CON**, OTA 0 mg/kg B.W. treated group; **OL**, OTA 1 mg/kg B.W. treated group; **OH**, OTA 3 mg/kg B.W. treated group.
